# Beryllium and its inorganic compounds – Addendum: Re-evaluation of the BAR: Assessment Values in Biological Material – Translation of the German Version from 2024

**DOI:** 10.34865/bb744041e9_4ad

**Published:** 2024-12-20

**Authors:** Julia Hiller, Wobbeke Weistenhöfer, Hans Drexler, Andrea Hartwig, MAK Commission

**Affiliations:** 1 Friedrich-Alexander-Universität Erlangen-Nürnberg. Institute and Outpatient Clinic of Occupational, Social, and Environmental Medicine Henkestraße 9–11 91054 Erlangen Germany; 2 Institute of Applied Biosciences. Department of Food Chemistry and Toxicology. Karlsruhe Institute of Technology (KIT) Adenauerring 20a, Building 50.41 76131 Karlsruhe Germany; 3 Permanent Senate Commission for the Investigation of Health Hazards of Chemical Compounds in the Work Area. Deutsche Forschungsgemeinschaft, Kennedyallee 40, 53175 Bonn, Germany. Further information: Permanent Senate Commission for the Investigation of Health Hazards of Chemical Compounds in the Work Area | DFG

**Keywords:** beryllium, biological reference value, BAR, exposure equivalents for carcinogenic substances, EKA, Urin, 7440-41-7, urine, 7440-41-7

## Abstract

The German Senate Commission for the Investigation of Health Hazards of Chemical Compounds in the Work Area (MAK Commission) summarised and re-evaluated the data for beryllium and its inorganic compounds regarding the recommended sampling times for biomonitoring, the derivation of exposure equivalents for carcinogenic substances (EKA) and the biological reference value (BAR) in urine. Only one new study reports data for beryllium in air and urine, but for the same beryllium concentration in air, the corresponding concentration in urine was lower than that given in two older publications. Considering the inconsistent data and questions on the validity of older analytical methods, the data base is not sufficient to derive EKA. The BAR was re-evaluated and a BAR of 0.02 µg/l urine was established based on the data for background exposure. Sampling should be conducted after a steady state has been reached. The data base is not sufficient for the evaluation of a BAR in blood.

**Table:** 

**BAR (2023)**	**0.02 µg beryllium/l urine**
	Sampling time: not fixed in the steady state; due to the long half-life, it may take several weeks to reach a steady state after taking up or resuming an activity at the workplace
**EKA**	**not established**
	
**MAK value (2003)**	**–**
Absorption through the skin	–
Carcinogenicity (2003)	Category 1
Sensitization (2002)	Sah

## Re-evaluation

Beryllium and its inorganic compounds were (re-)evaluated in 2002 and 2009. The available data, however, were not sufficient to derive exposure equivalents for carcinogenic substances (translated in Schaller 2005; Schaller et al. 2016). In 2009, a biological reference value (BAR) of 0.05 µg/l urine was derived (Schaller et al. 2016). The present addendum examines the possibility of deriving EKA and re-evaluates the BAR and the time of sampling. 

For the evaluation of measurement data on beryllium, it must be taken into account that especially in older studies (before the year 2000), analytical methods were frequently used which, according to more recent findings, are not sufficient in terms of sensitivity and specificity to reliably measure low beryllium concentrations (cf. Schaller 2005). For reliable biomonitoring of beryllium, the use of modern analytical methods such as primarily inductively coupled plasma mass spectrometry (ICP-MS) or optimised graphite furnace atomic absorption spectroscopy (GF-AAS) was therefore recommended (Schaller 2005; Schaller et al. 2016). Older studies that do not fulfil these criteria can therefore only be used to a limited extent for the evaluation of beryllium.

## Metabolism and Toxicokinetics

### Human data

In the case of occupational exposure to beryllium, inhalation is the main route of absorption. Data on the extent of possible deposition of beryllium in the lungs or inhalation bioavailability were not available in the last comprehensive assessments of the substance (cf. translated in Greim 2005; Schaller 2005). However, two older studies (Stiefel et al. 1980; Zorn et al. 1986) described biomonitoring data following accidental inhalation exposure. In addition to the questionable quality of the analytical data (e. g. measured values in non-exposed persons significantly higher than expected according to current standards; approx. 1 µg/l urine), some of the data in both studies are not comprehensible. Stiefel et al. (1980) reported on employees of a laboratory (n = 8) who were accidentally exposed to BeCl_2_, in which air concentrations of up to approx. 8 ng/m^3^ led to a simultaneous increase in the beryllium concentration in urine to about 4 times the initial value and a subsequent decline over approx. 10–15 days. Zorn et al. (1986) reported an accidental exposure to beryllium dust in 25 persons. One day after the exposure, the mean beryllium concentration in the serum was 3.5 ± 0.47 ppb (= µg/l) and seven days after the exposure it was 2.4 ± 0.3 µg/l. With the exception of the person with the highest internal exposure, all measurements after 2 to 8 weeks were within the reference range (assumed here to be up to 1 µg/l). Serum concentrations of 5.2 µg/l, 3.6 µg/l and approx. 2 µg/l were reported for the most severely exposed person 1 and 7 days and 6 months after exposure, respectively. Based on these data, the authors estimated a biological half-life in humans of approx. 2 to 8 weeks after a single inhalation exposure.

Since then, two further studies have been published with human elimination data following accidents. Aviv et al. (2018) reported on short-term inhalation exposure of employees to the radioisotope ^7^Be. In one volunteer, the lung retention of ^7^Be was determined up to 108 days using activity monitoring and the activity in four single void urine samples from the first 3 days was also measured. A two-phase elimination with a rapid first phase with a decay-corrected half-life between 0.4 and 1 day and a second, slower elimination with a half-life of approx. 109 days was determined for the lungs. A low activity of ^7^Be was also detectable in the urine samples. Thus, it can be assumed that at least a small proportion of the inhaled amount became systemically available. The highest activity concentration in urine was found one day after exposure (0.2 days after exposure, the measured activity in urine was 7.5 Bq/l; after 1.0 day 10.5 Bq/l; after 1.9 days 7.5 Bq/l and after 3.0 days 1.3 Bq/l). The rapid initial activity elimination phase from the lungs could possibly be due to a significant mucociliary clearance. Hiller et al. (2023) reported exposure to beryllium via direct tissue and blood entry due to an explosion trauma (explosion of a glass flask filled with 2.0 g beryllium in the hand of a laboratory employee with extensive soft tissue damage). From the medical follow-up over several years, including biomonitoring in urine (starting on day 2 after exposure) and blood (starting on day 147), a two-phase elimination kinetics in urine with half-lives of 117 and 666 days and a single-phase elimination kinetics in blood with a half-life of 103 days were determined. The maximum beryllium concentrations were 4.48 µg/l in urine on day 2 and 1.41 µg/l in whole blood on day 147. It should be noted that the exposure in the blood was only monitored with a delay of approx. 5 months. In addition, continued subacute exposure to beryllium via remaining splinter particles in the tissue cannot be ruled out. However, acute systemic exposure was assumed to be the most relevant route of exposure.

More recent field studies (with sufficiently sensitive analytics) also provide further data that can give indications of the temporal aspects of exposure monitoring in occupationally exposed collectives. Wegner et al. (2000) and Bounaurio et al. (2021) each described higher beryllium concentrations (not statistically significant) in urine in pre-shift samples compared to post-shift samples. In contrast, Morton et al. (2011) found a significantly higher beryllium excretion (47%) in urine samples obtained at the end of the week compared to corresponding samples from the beginning of the week. A multi-year study by Devoy et al. (2019) with data on beryllium concentrations in the air and urine of exposed employees from five different French companies analysed possible relationships between external and internal exposure (for a more detailed description, see Section Relationship between external and internal exposure).

### Data from animal experiments

Most of the available **animal data from non-inhalation exposure** (intravenous, intramuscular, subcutaneous, intraperitoneal) are not suitable to derive toxicokinetic data on elimination. Often only the distribution to different organs or tissues (Hard et al. 1977; Lindenschmidt et al. 1986) or data on whole-body retention (Furchner et al. 1973; Sakaguchi et al. 1993) are described. 

**Experimental animal data after inhalation exposure** show a long two-phase pulmonary clearance depending on the beryllium compound, the amount and the animal species investigated (Benson et al. 2000; Finch et al. 1990; Greim 2005; Haley et al. 1989, 1990; Rhoads and Sanders 1985; Sanders et al. 1975; Schaller 2005). 

There are also **experimental animal data on beryllium concentrations in the blood or urine following inhalation exposure**. Stiefel et al. (1980) described the first elimination of inhaled beryllium (^4^Be salt aerosols) in the urine of guinea pigs after 2 hours. The elimination maximum was 10 hours after the end of the 8 to 16-hour exposure. The concentrations in the urine returned to “normal levels” within 5 days after the peak concentration was reached. The increase in serum beryllium concentration paralleled the increase in urine; the time course of elimination in urine and serum is thus very similar. Finch et al. (1990) determined half-lives of elimination in urine of 103 and 58 days, respectively, after short-term (5–42 minutes) nasal exposure of dogs to BeO, depending on the manufacturing process. However, the low sensitivity and specificity of earlier analytical methods must be taken into account when evaluating these two studies. Muller et al. (2010) analysed urinary beryllium concentrations in mice after the first, second and third week of nose-only inhalation exposure and one week after the end of exposure (250 µg/m^3^ for 6 hours/day, 5 days/week, for 3 weeks) to various beryllium compounds (metallic Be, BeO, BeAl). The course of concentration in the urine during exposure and the extent of the decline after the end of exposure depended on the compound. 

## Exposure and Effects

### Relationship between external and internal exposure 

Due to the classification of beryllium as a category 1 carcinogen, the derivation of EKA was reviewed in 2003, but the available data were not considered sufficient (Schaller 2005). 

The following recent publications are used for the re-assessment of internal and external exposure: 

Morton et al. (2011) examined 167 employees from aluminium production and recycling. The beryllium concentrations in urine determined by ICP-MS were in the range from below the limit of quantification to 0.178 µg/l urine, with a mean of 0.0195 µg/l urine. Urine samples from the beginning and end of the week were available for 135 exposed employees. The exposed employees had 47% higher beryllium levels in their urine at the end of the week than at the beginning of the week. Bena et al. (2020) described a multi-year follow-up monitoring of employees of a waste incineration plant in Italy before and one and three years after commissioning of the plant. The beryllium concentration in the urine of 26 employees at the incinerator, for whom a complete history was available, tended to decrease over time (before commissioning: median: 0.19, 95^th^ percentile: 0.29 µg/l; after 3 years: median: 0.09, 95^th^ percentile: 0.13 µg/l). A control group of 9 administrative employees showed a comparable trend and similar values (before commissioning: median: 0.10, 95^th^ percentile: 0.37 µg/l; after 3 years: median: 0.08, 95^th^ percentile: 0.16 µg/l). Overall, the reported urine concentrations in this collective – especially before the plant was commissioned – tended to be higher than would be expected in occupationally unexposed collectives (compared to the BAR of 0.05 µg/l urine at the time). Bounaurio et al. (2021) analysed the urine of 40 metal workers/welders with regard to the beryllium concentration in pre-shift and post-shift samples, among other things. The beryllium concentrations in the urine were higher in the pre-shift samples (mean: 0.019, 95^th^ percentile: 0.039 µg/g creatinine) than in the post-shift samples (mean: 0.014, 95^th^ percentile: 0.020 µg/g creatinine). However, this difference was not statistically significant. 

Hulo et al. (2016) observed no statistically significant correlation between cumulative beryllium exposure and renal beryllium excretion in 30 employees exposed to beryllium (and 21 non-exposed control persons) from an aluminium production plant. The described statistically significant correlation of the cumulative beryllium exposure index with the beryllium concentration in the exhaled breath condensate does not necessarily reflect an internal exposure. 

In two of the three more recent field studies with simultaneous analysis of air and urine samples, no exposure could be quantified despite the use of ICP-MS (air samples) and GF-AAS methods (urine samples) in cases of questionable occupational exposure (Gerding et al. 2021; Godderis et al. 2005). In the study by Devoy et al. (2019), airborne beryllium exposures and the resulting urine levels were investigated over a period of 5 years in a total of 75 employees from five different French companies and a correlation model was derived. Due to optimised ICP-MS analytical methods, the sensitivity increased over the course of the study. Air samples were collected over an 8-hour working day using personal air pumps (flow rate 2 l/min); the limits of quantification were between 0.11 and 100 ng/filter, depending on the company. Parallel to the air measurements, urine samples were collected over at least five days, in some cases with several samples per day and for some employees also on two exposure-free days. 2600 urine samples with acceptable urine dilution were included in the evaluation. The limits of quantification for beryllium in urine were 0.1 µg/l in plant A (foundry for copper-beryllium (CuBe) compounds), 0.02 µg/l in plants B (aluminium-beryllium (AIBe) mechanical engineering company) and C (CuBe parts machining company) and 0.002 g/l in plants D (aluminium smelter) and E (aluminium foundry). As beryllium was below the limit of quantification (LOQ) in all urine samples in plants B and C and could be determined only in 3% and 12% of the air filters respectively (LOQ–2.5 ng/filter), the data from these two plants were not included in the further analyses. From plants A, D and E, 187 air measurements (range: < LOQ–7.58 µg/m^3^; geometric mean: 0.035 µg/m^3^) and 1350 urine samples (range: < LOQ–0.35 µg/g creatinine; geometric mean: 0.0047 µg/g creatinine) were available from 41 employees. There was a statistically significant correlation between the respective mean external and internal exposure. The concentration of 1 µg beryllium/m^3^ corresponded to an estimated beryllium excretion of 0.047 µg/g creatinine. Although an overall model showed that the workers-specific mean beryllium excretion in urine increased with statistical significance with the respective mean beryllium exposure in air, further kinetic analyses of the individual excretion profiles revealed no direct relationship to the concentration in the air. In particular, there was no excretion peak in the hours immediately after exposure and urinary excretion did not decrease during two-day exposure-free periods. The authors concluded that the determined urinary beryllium concentrations do not reflect recent external exposures and that the half-life of renal elimination is more than 48 hours. The derived correlation between urinary and air concentrations therefore reflects chronic exposure after a steady state has been reached.

## Re-evaluation of EKA 

There are currently only three studies available with air measurements and corresponding urine data (Apostoli and Schaller 2001; Devoy et al. 2019; Zorn et al. 1988). These show a very inconsistent picture regarding the relationship between air and urinary concentrations (see Table 1). Older studies used analytical methods with presumably inadequate sensitivity and specificity; in particular, the validity of the relationship described by Zorn et al. (1988) was regarded as very low (Schaller 2005).

**Tab. 1:** Beryllium concentrations in air and urine in occupationally exposed persons

**Beryllium**		**References**
**Air** **[µg/m^3^]**	**Urine** **[µg/l]**	
0.2 (2)	0.7 (7)	Zorn et al. 1988
0.2	0.12–0.15	Apostoli and Schaller 2001
1 *related to 0.2*	0.047 µg/g creatinine ≙ 0.056 µg/l^a)^ *0.011*	Devoy et al. 2019

^a)^ using the usual conversion factor of 1.2 g creatinine/l for mixed collectives when converting from creatinine to volume (Bader et al. 2020)

Due to the inconsistent data situation, it is therefore still not possible to derive sufficiently substantiated EKA. 

## Background exposure

The determination of background levels is only possible with sensitive analytical methods (limits of quantification in the range of 0.01 µg beryllium/l).

The following data on the background levels of beryllium in urine or blood in adults have been published since 2000 (Table 2):

**Tab. 2:** Concentrations of beryllium in urine or blood, plasma or serum in the general population and in collectives without occupational exposure to beryllium (studies from 2000 onwards; ICP-MS; values below the limit of quantification (LOQ) in italics (extrapolated from 3 times the limit of detection (LOD) if necessary)))

**Country**	**Collective (age)**	**Beryllium [µg/l]**			**References**
		**LOD/LOQ**	**Urine**	**Blood, plasma or serum**	
Germany	30 employees not exposed to metals age: MV 38.3 ± 11.5 years	LOD: 0.03 LOQ: no data	*median: < 0.03* *P95: no data* range: no data	–	Apostoli and Schaller 2001
Germany	63 persons of the general population age: ≥ 18 years	LOD: 0.004 LOQ: 0.014	*GM: 0.007* P95: 0.020 range: *LOQ*–0.028	–	Heitland and Köster 2004
France	100 healthy persons	LOD: 0.015 LOQ: 0.05	*median: 0.01* P95: 0.042 range (P5–P95): *0.008–0.042*	–	Goullé et al. 2005
		LOD: 0.03 LOQ: 0.10	–	plasma *median: 0.015* P95: 0.103 range (P5–P95): *0.015*–0.103 whole blood LOQ > 0.1			
Germany	87 persons of the general population 40 ♂, 43 ♀ age: 18–65 years	LOD: no data LOQ: 0.009	*GM: < LOQ* *P95: < LOQ* *range: < LOQ *	–	Heitland and Köster 2006 a
Germany	130 persons of the general population 50 ♂, 80 ♀ age: 18–70 years	LOD: no data LOQ: 0.008	–	whole blood *GM: < 0.008* P95: 0.015 range: *< 0.008–*0.04	Heitland and Köster 2006 b
Tokyo	78 healthy pregnant women (weeks 9 to 40 of pregnancy) age: 22–42 years, MV 32.1 years	LOD: 0.03 µg/g crea (= 0.024 µg/l) LOQ: no data	*GM: 0.031 µg/g crea* P95: no data range: *<0.03*–0.685 µg/g crea	–	Shirai et al. 2010
Italy	104 (A; Umbria) and 106 (B; Calabria) persons of the general population	LOD: 0.022 LOQ: no data	–	serum: *GM: 0.06 (A) / 0.05 (B)* P95: 0.09 (A + B) range: *0.02*–0.13 (A) and *0.02*–0.14 (B)	Bocca et al. 2010
UK	62 occupationally unexposed persons	LOD: 0.002 LOQ: 0.006	GM: 0.0095 (P90: 0.020) P95: 0.031^a)^ range: *< LOD*–0.044	–	Morton et al. 2011
UK	111 patients with kidney stones 77 ♂, 34 ♀ age: 21–85 years, median 51.5 years	LOD: 1.1 nmol/l (= 0.0099 µg/l) LOQ: no data (94.6% < LOD)	*median: < LOD* P95: no data *range (P2.5–P97.5): < LOD–2.7 nmol/24 h (0.0243 µg/24 h)*	–	Sieniawska et al. 2012
Belgium	1001 persons of the general population 460 ♂, 541 ♀ age: 18–80 years, MV 40.1 years	LOD: 0.007 LOQ: 0.022	*median: < LOD* *P97.5: < LOD* range: no data	–	Hoet et al. 2013
France	Employees not exposed to beryllium from companies in the aluminium production or optoelectrical industry number, sex, age not specified	LOD: 0.0006 LOQ: 0.0025	*GM: no data* *P95: no data* *range: < LOD*	–	Devoy et al. 2013
UK	132 persons of the general population 82 ♂, 50 ♀ age: 18–66 years	LOD: no data LOQ: 0.0006	280 samples (in some cases several samples per person) median: 0.0052 P95: 0.0116 range: no data	–	Morton et al. 2014
Spain	21 workers not exposed to beryllium from an aluminium production plant 10 ♂, 11 ♀ age: median 44 years (36–49 years)	LOD: no data LOQ: 0.0064	MV: 0.0152 µg/g crea P95: no data range: no data (90% > LOQ)	–	Hulo et al. 2016
France	2000 persons of the general population^b)^ 982 ♂, 1018 ♀ age: 20–59 years				Nisse et al. 2017
	1910 persons; 942 ♂, 968 ♀	LOD (urine): 0.0006 LOQ: no data	AM: 0.04 GM: 0.004 P95: 0.15 range (P10–P95): < LOD–0.15 (41.6% < LOD)
	1992 persons; 976 ♂, 1016 ♀	LOD (blood): 0.0004 LOQ: no data		whole blood AM: 0.02 GM: 0.003 P95: 0.09 range (P10–P95): < LOD–0.09 (42.7% < LOD)
USA	390 pregnant women (2^nd^–3^rd^ trimester) age: median 32.2 years	LOD: 0.04 LOQ: no data	*GM: 0.02* *P95: 0.11* range: no data (91.3% < LOD)	–	Kim et al. 2018
Spain	21 ♂, competitive athletes (A), 26 ♂, students (sedentary; B) age: MV about 22–23 years	LOD: 0.034 LOQ: no data	MV: 0.822 (A) and 0.116 (B) P95: no data range: no data	serum *MV: 0.09 (A)* *and 0.04 (B)* P95: no data range: no data	Maynar et al. 2018
USA	1335 ♀ from SWAN study^c)^ age: 45–56 years, MV 49.4 years	LOD: 0.04 LOQ: no data	*GM: not determined* (83.8% < LOD) *P95: 0.08* range (P5-P95): *< LOD–0.08*	–	Wang et al. 2019
Italy	35 employees of a waste incineration plant: (A) 9 ♂ in administration and (B) 26 ♂ employed in the plant before the plant was put into operation age: MV 42.8 years (A), 38.5 years (B)	LOD: 0.04 LOQ: no data	median: *0.10 (A)* and 0.19 (B) P95: 0.37 (A) and 0.29 (B) range: no data	–	Bena et al. 2020
Belgium	380 persons of the general population 178 ♂, 202 ♀ age: 18–70 years, MV 35.4 years	LOD: 0.03 LOQ: 0.08	–	whole blood *median: < LOD* *P97.5: < LOD* range: no data	Hoet et al. 2021
Germany	102 persons of the general population 38 ♂, 64 ♀ age: 19–66 years	LOD: no data LOQ: 0.02 (urine and whole blood)	*GM: < 0.02* *P95: < 0.02* *range: < 0.02 *	whole blood *GM: < 0.02* *P95: < 0.02* *range: < 0.02 *	Heitland and Köster 2021
		LOD: no data LOQ: 0.004 (serum)	–	serum *GM: < 0.004* *P95: < 0.004* *range: < 0.004 *			
Germany	20 office workers from electrical waste recycling companies 15 ♂, 5 ♀ age: 27–59 years, MV 50.6 years	LOD: 0.03 LOQ: 0.09	*GM: 0.02* *P95: 0.05* *range: 0.02–0.05 *	–	Gerding et al. 2021
Italy	13 ♂ unexposed employees of a metalworking company age: 20–59 years, MV 44.9 years	LOD: 0.03 LOQ: 0.09	*MV: 0.01 µg/g crea* *P95: 0.015 µg/g crea* *range (P5–P95): 0.007–0.015 µg/g crea^d)^*	–	Buonaurio et al. 2021
Germany	77 persons of the general population 34 ♂, 43 ♀ age: 19–78 years, MV 45.8 years	LOD: no data LOQ: 0.003	*GM: no data* *P90: < LOQ* *P95: < LOQ^e)^* range: *< LOQ*–0.005 (98% < LOQ)	–	Schmied et al. 2021
China	63 office employees without metal exposure 46 ♂, 17 ♀ age: MV 39.3 ± 11.4 years	LOD: 0.007 LOQ: no data	*MV: 0.0178* P95: 0.0245 range: *0.0133–*0.0299	serum MV: 0.0264 P95: 0.0486 range: *0.0038*–0.0584	Liu et al. 2021
Norway	757 healthy persons of the general population^f)^ 375 ♂, 382 ♀ age: 49–66 years, MV 57.4 years	LOD: 0.02 LOQ: no data	–	whole blood: MV: 0.035 *median: < 0.02* P90: 0.088 range: *< 0.020*–0.359^h)^	Syversen et al. 2021
Norway	1011 persons of the general population^g)^ 506 ♂, 505 ♀ age: 20–91 years, MV 50.0 ± 17.6 years	LOD: 0.0096 LOQ: no data	–	whole blood: *GM: < 0.0096* *P95: 0.0122* *range: < 0.0096–0.0260*^h)^	Simić et al. 2022

^a)^ Morton 2023

^b)^ formerly heavily industrialised area

^c)^ longitudinal study on midlife women

^d)^ values calculated with LOD/2 => cannot be used meaningfully, as probably below LOQ; no data on creatinine levels in urine

^e)^ Schmied 2023

^f)^ sub-collective 1 from HUNT3 study: The Nord-Trøndelag Health Survey from 2006-2008; initially selected for neuroimaging study;

^g)^ sub-collective 2 from HUNT3 study, not identical with the persons from Syversen et al. (2021)

^h)^ differences between the results of Syversen et al. (2021) and Simić et al. (2022) most likely due to more stable analysis in the study of Simić (Syversen 2023)

AM: arithmetic mean; crea: creatinine; GM: geometric mean; MV: mean value; P2.5, P5, P10, P90, P95, P97.5: 2.5^th^, 5^th^, 10^th^, 90^th^, 95^th^, 97.5^th^ percentile

## Re-evaluation of the BAR

Comparatively few studies are available on beryllium concentrations **in blood and serum**; a BAR for beryllium in blood could therefore not be evaluated.

With regard to the re-evaluation of the BAR **in urine**, preference was given to data from the German population and subsequently from the European area. Only studies in which the limit of quantification was ≤ 0.05 µg/l (corresponding to the previous BAR) or the limit of detection was ≤ 0.02 µg/l were considered. Table 3 shows an overview of all studies with sufficient analytical sensitivity and specificity and representative collectives for the derivation of a BAR, in which the 95^th^ percentile of the beryllium concentration in urine was given. 

**Tab. 3:** Assessment-relevant studies on the background levels of beryllium in urine in the general population

**Number of persons**	**95^th^ percentile beryllium [µg/l urine]**	**References**
European studies		
64	0.02	Heitland and Köster 2004
62	0.031^a)^	Morton et al. 2011
132	0.012	Morton et al. 2014
1910	0.15	Nisse et al. 2017
European studies with 95^th^ percentile < LOQ		
100	< LOQ (0.05) *0.042*^b)^	Goullé et al. 2005
87	< LOQ (0.009)	Heitland and Köster 2006 a
111	~ LOD (0.0099) (94.6% < LOD)	Sieniawska et al. 2012
1001	< LOD (0.007)	Hoet et al. 2013
no data	< LOD (0.0006)	Devoy et al. 2013
102	< LOQ (0.02)	Heitland and Köster 2021
65	< LOQ (0.003)	Schmied et al. 2021
Non-European studies		
63 (China)	0.0245 (> LOQ)	Liu et al. 2021

^a)^ Morton 2023

^b)^ value from publication, but < LOQ

LOD: limit of detection; LOQ: limit of quantification

There are four studies on background levels of beryllium in urine in Germany (Heitland and Köster 2004, 2006 b, 2021; Schmied et al. 2021). The 95^th^ percentile was < 0.02 µg beryllium/l in all cases. However, the 95^th^ percentile was reliably quantifiable only in one study, i. e. was above the limit of quantification. 

Four European studies are available reporting a 95^th^ percentile above the limit of quantification. With the exception of the French study by Nisse et al. (2017), the 95^th^ percentiles are between 0.01 and 0.03 µg/l. This is in good agreement with the other eight supporting studies with information on urine concentrations, five of which have a 95^th^ percentile of < 0.01 µg/l and the rest are compatible with a 95^th^ percentile between < 0.02 and < 0.05 µg/l. Possible explanations for the deviation of the data from Nisse et al. (2017) from the other data could be that the people analysed there came from an area that was previously heavily polluted by industry. On the basis of these data and with special consideration of the data from Germany, a 

**BAR of 0.02 µg beryllium/l urine**

is derived.

## Interpretation

The data on the absorption and excretion of beryllium depended on the dose, the solubility and the chemical form of the beryllium compounds. However, in the case of chronic exposure the data do not suggest any significant influence of higher short-term exposure. The long half-lives in urine and blood of up to more than 100 days described in several studies are also consistent with this. Samples should therefore be taken when beryllium is in steady state. Due to the long half-life, it may take a long time (several weeks) after (re-)commencement of the activity until the steady state is reached.

With regard to short-term or single exposures, in particular via inhalation, which is the primary route of uptake in the occupational context, systemic exposure can be expected to be detectable with a delay of several hours due to necessary resorption processes. The maximum elimination in urine in this case would most likely be expected in the range of 10 to 24 hours after the end of exposure (Aviv et al. 2018; Stiefel et al. 1980; Zorn et al. 1986).

The BAR refers to normally concentrated urine in which the creatinine concentration is in the range of 0.3–3 g/l. For urine samples outside the above limits, it is generally recommended to repeat the measurement in normally hydrated persons (Bader et al. 2016).
